# DNP-KLH Yields Changes in Leukocyte Populations and Immunoglobulin Isotype Use with Different Immunization Routes in Zebrafish

**DOI:** 10.3389/fimmu.2015.00606

**Published:** 2015-12-01

**Authors:** Heather Weir, Patricia L. Chen, Thaddeus C. Deiss, Natalie Jacobs, Mary B. Nabity, Matt Young, Michael F. Criscitiello

**Affiliations:** ^1^Comparative Immunogenetics Laboratory, College of Veterinary Medicine and Biomedical Sciences, Texas A&M University, College Station, TX, USA; ^2^Department of Veterinary Pathobiology, College of Veterinary Medicine and Biomedical Sciences, Texas A&M University, College Station, TX, USA; ^3^Department of Science, A&M Consolidated High School, College Station, TX, USA

**Keywords:** zebrafish, mucosal, immersion immunization, BAFF, IgZ

## Abstract

Distinct methods are required for inducing mucosal versus systemic immunity in mammals for vaccine protection at the tissues most commonly breached by pathogens. Understanding of mucosal immunization in teleost fish is needed to combat aquaculture disease, understand emerging ecological threats, and know how vertebrate adaptive immunity evolved. Here, we quantitatively measured expression levels of IgM as well as the teleost mucosal immunoglobulin, IgZ/IgT, in zebrafish given an antigen systemically via intraperitoneal (i.p.) injection or mucosally via bath immersion. Both immunoglobulin isotypes and the B cell activating factor gene transcription was induced in fish injected with antigen as compared to saline injected or antigen immersed fish, though these failed to reach statistical significance. Here we provide additional reference hematology for this model species. Differential blood counts revealed a greater lymphocyte percentage in both i.p. and immersed fish, with increase in large lymphocyte counts and decrease in neutrophils. These humoral adaptive gene transcription and cytological data should provide a foundation for more studies connecting immunology in this dominant developmental and genetic fish model to other species where mucosal immunization is of greater commercial importance.

## Introduction

The adaptive immune system of jawed vertebrates with the hallmark characteristics of specificity and memory is mediated by lymphocytes [reviewed in Ref. (1)]. The clonal expansion of lymphocytes specific for an antigen of a pathogen is the basis for the preemptive engineering of immune repertoires through vaccination, the most powerful tool for global public health.

Vaccination is also the best instrument for combating disease in high-density finfish aquaculture. Most pathogens breach mucosal barriers and thus mucosal immunity is needed against most infectious organisms, yet most aquatic vaccines are delivered by labor-intensive injection (2). It is predicted that for fish, as in mammals, route of delivery will be important in the success or failure of mucosal vaccination (3). The zebrafish (*Danio rerio*) has for decades been the ectothermic vertebrate species for genetic dissection of vertebrate development and is now becoming a choice model for interrogation of pathologic mechanisms from cancer to infection (4). Although some baseline hematology and leukocyte characterization exists in zebrafish (5–7), powerful technologies such as *in vivo* imaging (8) have advanced the zebrafish model while much vaccinology has largely proceeded in fish of aquacultural importance. This has left large gaps in the fundamental immunology of the most prominent teleost fish species in biomedicine.

B lymphocytes produce immunoglobulins (Ig) for adaptive humoral immunity from sharks to mammals (9). While mammals possess five functionally distinct Ig heavy chain isotypes (IgM, IgD, IgG, IgA, and IgE), teleost fish have only three [IgM, IgD, and IgZ (10–13)]. So far, IgZ is an isotype restricted to bony fish, and sequence characteristics (10), gut localization and functional work (14) have suggested that it is a dedicated mucosal isotype (15), functionally analogous but not orthologous with IgX/A of tetrapods (16). Whether fish B cells produce IgM/D or IgZ can be determined by instructive IgH locus organization. In some fish (including zebrafish) shared Vs rearrange with D segments dedicated to IgZ or IgM/D to determine isotype lineage, whereas in others (such as tuna) D segments are shared and the D join to J segments dedicated to either isotype appear to decide commitment (17). This teleost mucosal isotype was given the name IgT in trout (10), but IgZ in zebrafish (12), so we will use that appellative here. IgZ does not appear to be used by all teleost fish, however, as at least catfish and medaka show no evidence of it genomically, transcriptionally and serologically (13, 18).

At least four mucosal immune compartments have been identified in bony fish: gut associated lymphoid tissue (GALT) (19), skin associated lymphoid tissue (SALT) (20), nasal associated lymphoid tissue (NALT) (21), and gill associated lymphoid tissue (GIALT) (22) sometimes containing interbranchial lymphoid tissue (ILT) (23). These join the spleen and pronephros as secondary lymphoid tissues, although the architecture of these latter two is better defined into B and T cell zones (24). These multiple sites for potential initiation of adaptive immune responses in fish have heightened hopes in the aquaculture community for new methods of mucosal immunization.

In the present study, we set out to characterize the basic cellular and humoral adaptive immune response to a routine hapten-protein carrier [Dinitrophenyl-conjugated keyhole limpet hemocyanin (DNP-KLH)] antigen delivered via i.p. injection or mucosal bath immersion to adult zebrafish. We assayed lymphocyte percentages in peripheral blood, spleen transcript levels of IgM, both zebrafish IgZ isotypes (25), and a critical cytokine in B cell survival, proliferation, maturation and differentiation: the B cell activating factor (BAFF) that has been characterized from zebrafish (26). In addition to providing additional reference cytological and molecular values for future immunization trials, this work provides leukocyte morphological characterization for this model species.

## Materials and Methods

### Animals and Sample Harvest

Outbred zebrafish (*D. rerio*) were obtained at a local pet store and quarantined in filtered aquaria in the biotechnology laboratory at A&M Consolidated High School until health and immunologic maturity was assured (27). Fish were maintained through termination at a high school without an animal institutional care and use committee, so work followed the guidelines of the American Association of Laboratory Animal Science for the Use of Animals in Precollege Education, found here: https://www.aalas.org/about-aalas/position-papers/use-of-animals-in-precollege-education. Three groups (control, mucosal, and injection) of 15 fish each were maintained in separate 10 gal aquaria with external power filters, aeration, and thermostatic heaters. Three fish were lost from the injection group in the first 2 weeks of the experiment. At the conclusion of the experiment, fish were euthanized by immersion in 500 mg/L MS-222 [tricaine methanesulfonate (Finquel), Argent Laboratories, Redmond, WA, USA], and blood was collected in a microhematocrit tube from caudal vein after tail amputation at the caudal peduncle. Fish were then dissected for immediate harvest of spleen into RNALater (Qiagen, Valencia, CA, USA).

### Immunizations

Fish in all three groups were anesthetized before each treatment by immersion in a 50 mg/L solution of MS-222, and observation until righting behavior was compromised (5–20 s) but before loss of buoyancy. DNP-KLH (CalBiochem, San Diego, CA, USA) was used as an experimental antigen in these trials without any adjuvant. Fish in the injection group received 50 μL 1 mg/L DNP-KLH delivered to the abdominal cavity with a 30-gage needle, control group fish received a similar injection with 50 μL phosphate buffered saline. Fish in the mucosal group were immersed in a 35 mg/L DNP-KLH solution for 15 min after anesthesia. Fish received injection, control injection, or mucosal treatment once weekly for 4 weeks. These doses and immunization schemes were adapted from studies with DNP-KLH in other teleosts (28–31). All fish were observed in a well-oxygenated 2 gal recovery tank after treatment before returning to 10 gal aquaria.

### Hematology

Blood smears were made immediately after blood collection using a drop of blood spotted on one microscope slide and drawn with the edge of another to obtain one to two feathered edged smears. Smears were stained with a modified Wright’s/Giemsa stain and a 100-cell leukocyte differential was performed for each fish. Images were captured using an Olympus BX51 microscope with a 100× oil immersion objective, Diagnostics Instruments camera, and SPOT Advanced imaging software (Diagnostic Instruments, Sterling Heights, MI, USA).

### Quantitative PCR

Total RNA was purified from RNALater preserved spleen using the RNAeasy minikit (Qiagen) according to the manufacturer’s instruction. The quantity and quality of the RNA was assayed by NanoDrop 2000c spectrophotometry (Thermo Scientific, Wilmington, DE, USA) and Agilent 2100 Bioanalyzer (Agilent, Santa Clara, CA, USA), respectively. Superscript III First Strand Synthesis kit (Life Technologies, Grand Island, NY, USA) was used for cDNA production using a 1:1 ratio of oligo dT and random hexamer primers.

Primers were designed for the constant domain genes of zebrafish IgM, IgZ1 and IgZ2, BAFF and the housekeeping gene Rpl13α (Table S1 in Supplementary Material) and checked for the production of a singular amplicon by traditional PCR and sequenced for verification. Real-time PCR reactions were performed with 50 μg cDNA, 1.25 mM MgCl_2_, and HOT FIREPol High Resolution Melt mix (Solis BioDyne, Tartu, Estonia) and EvaGreen (Mango Biotechnology, Mountain View, CA, USA) using a Roche LightCycler 480 and a three-step thermal cycling program: one cycle at 95°C for 5 min, then 45 cycles of 95°C for 10 s, 60°C for 5 s, and 72°C for 5 s. Roche LightCycler software was utilized for raw data acquisition and calculation of C_t_ (threshold cycle) values.

Changes in gene expression were estimated using the 2^−ΔΔCt^ method (32), with Rpl13a used as the housekeeping gene for all experiments. The fold changes in gene expression were calculated with respect to the expression level of the genes in the PBS injected control fish for the data in the publication and the day 0 controls of each group for Figure S1 in Supplementary Material.

### Statistical Analysis

The mean expression and SD of the lymphocyte percentages and gene expression were calculated using the summarySE function from Rmisc in R (33, 34). Analysis for variance of means (ANOVA) to identify any statistical relevance was performed in the base R package, any statistically relevant values were subjected to the *post hoc* TukeyHSD to corroborate ANOVA findings (34). Graphs with error bars were created using the ggplot2 package in R (35).

## Results

In order to explore the effects route of antigen exposure have in humoral adaptive immune responses elicited in zebrafish, DNP-KLH was given either through i.p. injection or mucosal bath immersion four times at 1-week intervals to adult zebrafish, and they were euthanized 1 week after the last treatment. In addition to monitoring levels of B cell gene expression via molecular techniques, we wanted to assess changes in peripheral blood lymphocyte levels. We started with careful leukocyte characterization to complement the available information in this species (36).

### Zebrafish Leukocyte Identification

**Figure 1:**
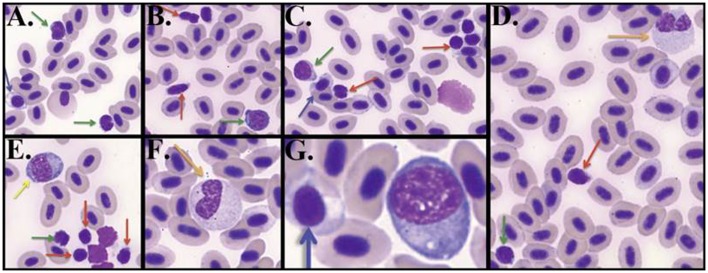
**Zebrafish hematology**. Wright/Giemsa stained zebrafish blood smears (**A–C**) Differentiating small lymphocytes (green arrows) from thrombocytes (red arrows). Immature reticulocytes are also seen (blue arrows) **(D–G)**. Neutrophils (orange arrows) are typically seen with bilobed nuclei, whereas large mononuclear cells (yellow arrow) have a round nucleus. Blood smear images were captured under 1000× total magnification with oil.

Unlike mammalian blood smears, fish exhibit nucleated erythrocytes and thrombocytes instead of platelets (37). Lymphocytes contained a small amount of blue cytoplasm containing granules, had round nuclei that could be indented and, in contrast to thrombocytes, displayed a stippled or smudged chromatin (Figure 1). Categorization of thrombocytes was aided by their scant amount of clear to light blue cytoplasm and indistinct cell borders, “glassy” chromatin pattern, round to elongate nucleus, and frequent presence in clumps. Immature erythrocytes on the other hand contained ample cytoplasm. Large mononuclear cells were evident that are presumed large lymphocytes containing a moderate amount of dark blue cytoplasm, often with vacuoles and a round or irregular nucleus. Small lymphocytes contained similarly dark stained nucleus but with less cytoplasm. Neutrophils contained a light pink to light blue cytoplasm and usually a bilobed or banded nucleus, although some neutrophil nuclei were round.

### Differential Leukocyte Counts

**Figure 2:**
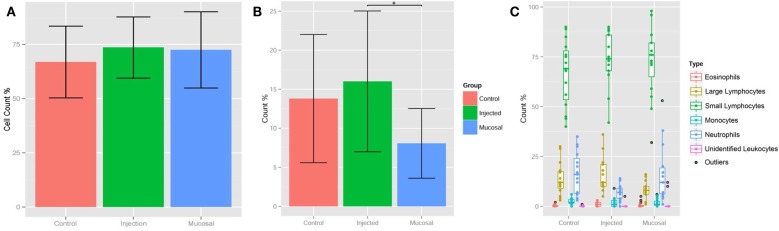
**Lymphocyte percentages shift upon immunization**. **(A)** Bar graphs displaying percentages of small lymphocytes only and **(B)** large lymphocytes in peripheral blood at termination of 4-week immunization experiment. **(C)** Box and whisker plots of all cell type percentages. The box extends from first quartile to third quartile, the line in the box is the median, and whiskers extend to 1.5 times the inter-quartile range, outliers are points outside this range. Asterisks *p* < 0.01.

Having standardized our differentiation of lymphocytes and granulocytes, we could perform differential blood cell counts on the terminal bleeds of the zebrafish in the three experimental groups (Table 1 and Datasheet S1 in Supplementary Material). Comparing the percentages of small lymphocytes, a slightly higher percentage was found in both the mucosal and i.p. immunization groups compared to the control group, but this did not reach statistical significance (Figure 2A). The percentage of large lymphocytes in peripheral blood was increased significantly in the i.p. injected fish over the mucosal fish (Figure 2B); however, neither of these was significantly increased over control PBS injected. The relative percentages of other cell types remained largely consistent between the three treatments; however, the neutrophil percentage was compromised when lymphocytes expanded (Figure 2C). This dip in neutrophil percentages could simply indicate an increase in lymphocyte percentages, without a corresponding change in absolute neutrophil number. However, it could also reflect an actual decrease in neutrophil number due to localized diapedesis or decreased production.

**Table 1:** **Differential blood counts (lymphocyte summary)**.

Fish	Control			Injection			Mucosal		
	SmLym (%)	LgLym (%)	Total (%)	SmLym (%)	LgLym (%)	Total (%)	SmLym (%)	LgLym (%)	Total (%)
#1	51	18	69	88	12	100	82	2	84
#2	56	30	86	80	10	90	86	7	93
#3	68	11	79	75	11	86	98	1	99
#4	72	10	82	42	29	71	96	2	98
#5	85	8	93	73	16	89	72	13	85
#6	80	14	94	89	8	97	73	9	82
#7	69	12	81	74	12	86	76	7	83
#8	89	3	92	71	18	89	71	6	77
#9	62	12	74	66	21	87	82	9	91
#9(R)				68	22	90			
#10	45	12	57	90	5	95	59	10	69
#11	44	22	66	54	36	90	55	6	61
#12	75	5	80	86	8	94	32	15	47
#13	40	29	69				49	8	57
#14	76	17	93				78	16	94
#15	90	4	94				78	10	88

### Immunoglobulin and BAFF qPCR

**Figure 3:**
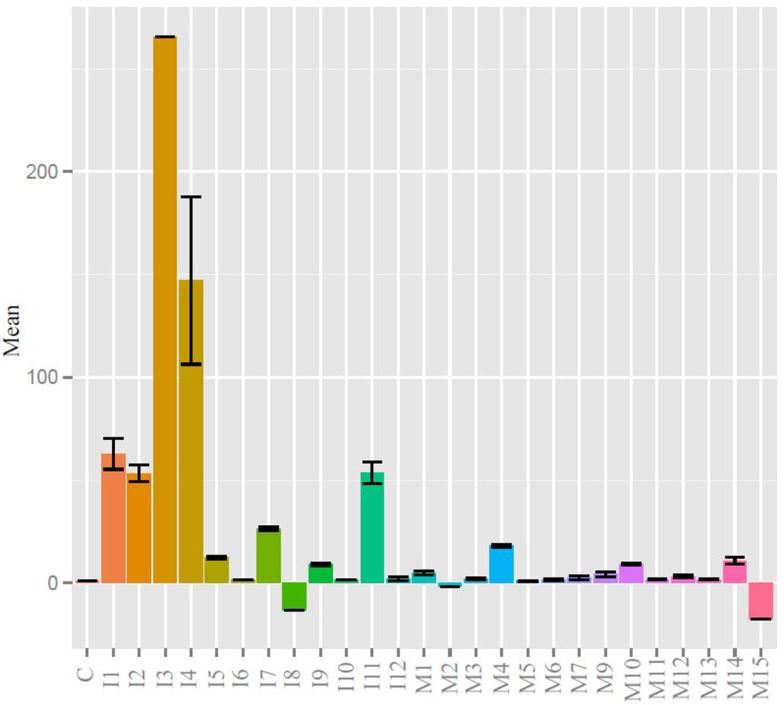
**IgM levels were raised in injected fish compared to control and mucosal, but not to statistical significance**. All statistically relevant differences observed via ANOVA and *post hoc* TukeyHSD indicated by asterisks *p* < 0.01.

Quantitative real-time PCR was used to measure gene expression of the major immunoglobulin isotypes and BAFF. BAFF binding to the BAFF receptor activates both the classical and non-canonical NFκB pathways generating signals critical for B cell formation and maintenance (38). BAFF was chosen as a transcription factor whose expression was indicative of humoral adaptive immune induction as it and IL-4 have been shown to play a critical role in zebrafish IgM responses (39). More BAFF, IgM, IgZ1, and IgZ2 were expressed in the injected fish than the control or mucosally immunized, yet wide individual variation prevented any significance (Figure S1A in Supplementary Material). The expression profiles of products of the two IgZ constant genes appeared to be in lock-step in the three groups. When the mean immunoglobulin expression differences were analyzed, the injection group was statistically greater than that of the control and mucosal groups (Figure S1B in Supplementary Material). We analyzed individual fish at the experimental terminus compared to PBS injected controls. IgZ was too low to detect for most samples but IgM values are shown in Figure 3. DNP-KLH injected fish had generally higher responses than control and mucosally stimulated fish, but individual variation was high and comparisons between groups did not reach statistical significance.

## Discussion

### Hematology

We provide here fundamental hematology for zebrafish blood cells to complement the growing body of molecular tools for marking immune cells. The lymphocyte percents in peripheral blood averaged 47–100% in this study, a wider range than the 71–92% that has previously been published (6). Eosinophils of zebrafish have been distinguished from neutrophils via *gata2* expression (40), although we did not see the high levels of these cells from caudal bleeds that have been observed in pronephros and peritoneal cavity. Dendritic antigen presenting cells have been identified in zebrafish with peanut agglutinin and are able to activate T cells in an antigen-dependent manner (41). Scavenger receptor CD36 was expressed much lower in macrophages and neutrophils of zebrafish and carp than what is observed in human (42). Diverse staining approaches have been used to account for changes in immune cell number in zebrafish as well, as granulocytes have been colored metachromatically with Toluidine Blue and enumerated (43).

Although we saw an increase in large lymphocytes in the injected fish over mucosally exposed ones, the mucosally treated large lymphocyte numbers were less than the control PBS injected. This suggests that the injection itself has some immunostimulatory effect, possibly via introduction of mutualistic bacteria from the external mucous layer into the body cavity. These expanded percentages of large lymphocytes could represent plasmablasts with expanded Golgi bodies. The shift toward higher percentages of large and small lymphocytes in the DNP-KLH-injected fish corresponds to a decrease in neutrophil percentage, although they may not be dropping in actual cell numbers.

### Ig Isotypes

Whereas others have found differential expression of the two IgZ subclasses, including more IgZ2 up-regulation in spleen with LPS stimulation (25), we found parallel expression in our study. It remains to be seen whether the significant primary amino acid differences in the constant regions of these two antigen receptors translate into physiological differences, as constant regions of other vertebrate antigen receptors have been found with significant diversity due to polygeny [e.g., 13 rabbit IgA genes (44)] or polymorphism [e.g., high allelic polymorphism at teleost α and β T cell receptor constant domain genes (45, 46)] without clear functional distinction. More work is needed to determine if IgZ2 functions on a distinct set of B cells, and if so where they are activated. As in other vertebrates (47), we expect non-translatable “sterile” transcripts from the IgH locus to contribute to the amplicons measured by constant region qPCR contributing to an inflation of signal compared to protein. Interbranchial lymphoid tissues have been proposed as a secondary mucosal tissue in salmon where IgT-expressing B cells clonally expand (48). The polymeric Ig receptor (pIgR) is expressed in the gut (14) and skin (49) of some teleosts where it is needed for transport of IgM and IgT across mucosal barriers. In zebrafish, a great many genes related to pIgR have been identified that bind phospholipids, and some that appear to be inhibitory membrane bound receptors (50).

While functional distinction in immunoglobulins is routinely associated with the heavy chain there are cases of non-random light chain expression of functional distinction (51–53). In lower vertebrates where more immunoglobulin light chain isotypes exist (54), we should expect to find more evidence of light chain isotype function (Natalie Jacobs and Michael F. Criscitiello, manuscript in preparation). The multiple light chain genomic organizations of the zebrafish would make this an interesting model to further explore relationships between immunoglobulin light chain isotypes, genomic organization, paratope construction, and heavy chain isotype (55, 56). Use and light chain dimerization with IgD also deserves more study in zebrafish (57).

### Routes of Immunization in Zebrafish

Most immunization studies in adult zebrafish have utilized i.p. or i.m. injection in adults and embryos of 1–3 days postfertilization are micro-injected directly into posterior blood island or Duct of Cuvier (58). But skin exposure to a gram positive and gram negative bacteria both gave similar changes in innate immune gene transcription profiles (59, 60), thus we wanted to focus on humoral adaptive immunity to a classic protein hapten-carrier complex in this study. One-day-old zebrafish have been immersion infected with *Edwardsiella tarda* and *Flavobacterium columnare*; however, high mortality rates and individual variation are an issue in such studies as in this one.

Other studies have targeted particular genes crucial to zebrafish host defense that may lend to deliverables for aquaculture. The proliferation-inducing ligand APRIL (also known as tumor necrosis factor ligand superfamily member 13 or TNFSF13) has been identified in zebrafish and it promotes the survival of fish lymphocytes in a dose-dependent fashion (61). APRIL is recognized by the transmembrane activator and CAML interactor (TACI). Knockdown of superoxide dismutase 2 in zebrafish causes increased susceptibility to *Pseudomonas aeruginosa* and decreased numbers of phagocytes (62).

Future studies would benefit from untreated controls including PBS or mock injections, to control for flora in the tank water and slime coat that could be introduced in the control injections and induce potent immune responses (63). Additionally, other antigens should be explored and additional time points <1 week and longer than those monitored in this study should be assayed. Monitoring of other immune tissues such as MALT, pronephros and gill in addition to a non-immune tissue would likely reveal more insights into the adaptive humoral response in this fish. Importantly, we cannot be sure that immunization at mucosal sites will induce a systemic response measurable in the spleen (64).

### Future Directions

In order for the translation of this work into effective mucosal vaccines for fish such studies need to be complemented with characterization of the nature, location, and requirements of memory IgT producing plasma cells and memory B cells, as well as a better understanding of what adjuvants or innate stimulants will enhance specific immunity. Fish have successfully been orally immunized to iridovirus by being fed rice callus producing recombinant antigen (65), and these exciting advances need to be followed up to see if protection is afforded at all mucosal surfaces. Furthermore, all sites of systemic and mucosal antibody production must be elucidated and characterized in fish (66), noting that they may be different in different taxanomic groups. Tools for transcriptomic analysis are becoming less script writing intensive, as GeneTiles recently facilitated a comparison between *Staphylococcus epidermidis* and *Mycobacterium marinum* infected zebrafish embryos (67). Attention should also be given to the effect of passive immunity on the embryo, as pathogen specific IgM at least is transferred to the egg and can protect the developing zebrafish against *Aeromonas hydrophila* (68).

## Conclusion

In conclusion, injection of hapten-conjugated protein antigen (DNP-KLH) in the absence of adjuvant resulted in lower neutrophil percentages and higher large lymphocyte percentages, and elicited B cell transcription factors and antibody gene up-regulation, but static immersion into the same antigen did not yield similar result in zebrafish. Importantly, this study did not confirm antibody transcript levels with protein data, and cells and message were only assayed from peripheral blood and spleen, ignoring mucosal associated lymphoid tissue. Several variables should be explored in future experiments including titrating dose of both injected and immersion antigen, a different analgesic such as low temperature instead of MS-222 (69), diet (70), and other antigens. Although the magnitudes of T-dependent secondary and T-independent primary responses in the teleost catfish were found to be largely independent of temperature, the primary response to DNP-KLH was found to be suppressed at lower temperatures (31). Eventually repertoire characterization of the responses elicited by zebrafish immunizations will be enlightening, as spectratyping and robust repertoire sequencing analysis of the response in trout spleen to systemic viral infection found complex public and private IgM clonal expansions, some IgT, and little IgD (71).

## Conflict of Interest Statement

The authors declare that the research was conducted in the absence of any commercial or financial relationships that could be construed as a potential conflict of interest.
