# Supplementary material for: DNP-KLH Yields Changes in Leukocyte Populations and Immunoglobulin Isotype Use with Different Immunization Routes in Zebrafish
Source: Front Immunol. 2015 Dec 1;6:606. doi: 10.3389/fimmu.2015.00606 (PMC4664633; doi:10.3389/fimmu.2015.00606)
Supplement: Supplementary file 1 [file Table_1.DOCX]

| Supplemental Table I. Primers used in PCR | | | | | |
| --- | --- | --- | --- | --- | --- |
| Primer Name | For/Rev | Domain | Size | Sequence | Priming Site |
|  |  |  |  |  |  |
| MFC464 | F | IgMC1 | 218 | 5’-CATATACGAGTGAGAAAAAGCGACTG-3’ | HIRVRKSDW |
| MFC465 | R | IgMC2 |  | 5’-TATTGTTTAGGCGAAAACCGCCGG-3’ | RRFSPKQY |
| MFC466 | F | IgZC1 | 296 | 5’-AACGTCACCCAGCATTCTACAGC-3’ | NVTQHSTA |
| MFC467 | R | IgZC2 |  | 5’-CCATTCATGTTCAGTTTGTACTCCAG-3’ | LEYKLNMNG |
| MFC468 | F | IgZ2C1 | 249 | 5’-AGCCTGACCAATTTTATCCAATACCC-3’ | SLTNFIQYP |
| MFC469 | R | IgZ2C2 |  | 5’-TTTGGGGTAGAAATCCTCCATAACAC-3’ | CVMEDFYPK |
| MFC523 | F | BAFF | 136 | 5’-CCTCCTCTTCGCTGTCTGTG-3’ | TSSSLSV |
| MFC524 | R | BAFF |  | 5’-TCTGCCTCGTTCACAGGTTC-3’ | EPVNEAE |
| MFC462 | F | RPL13α | 148 | 5’-TCTGGAGGACTGTAAGAGGTATGC-3’ | WRTVRGM |
| MFC463 | R | RPL13α |  | 5’-AGACGCACAATCTTGAGAGCAG-3’ | PAALKIVR |
