# Supplementary figures and images for: DNP-KLH Yields Changes in Leukocyte Populations and Immunoglobulin Isotype Use with Different Immunization Routes in Zebrafish
Source: Front Immunol. 2015 Dec 1;6:606. doi: 10.3389/fimmu.2015.00606 (PMC4664633; doi:10.3389/fimmu.2015.00606)

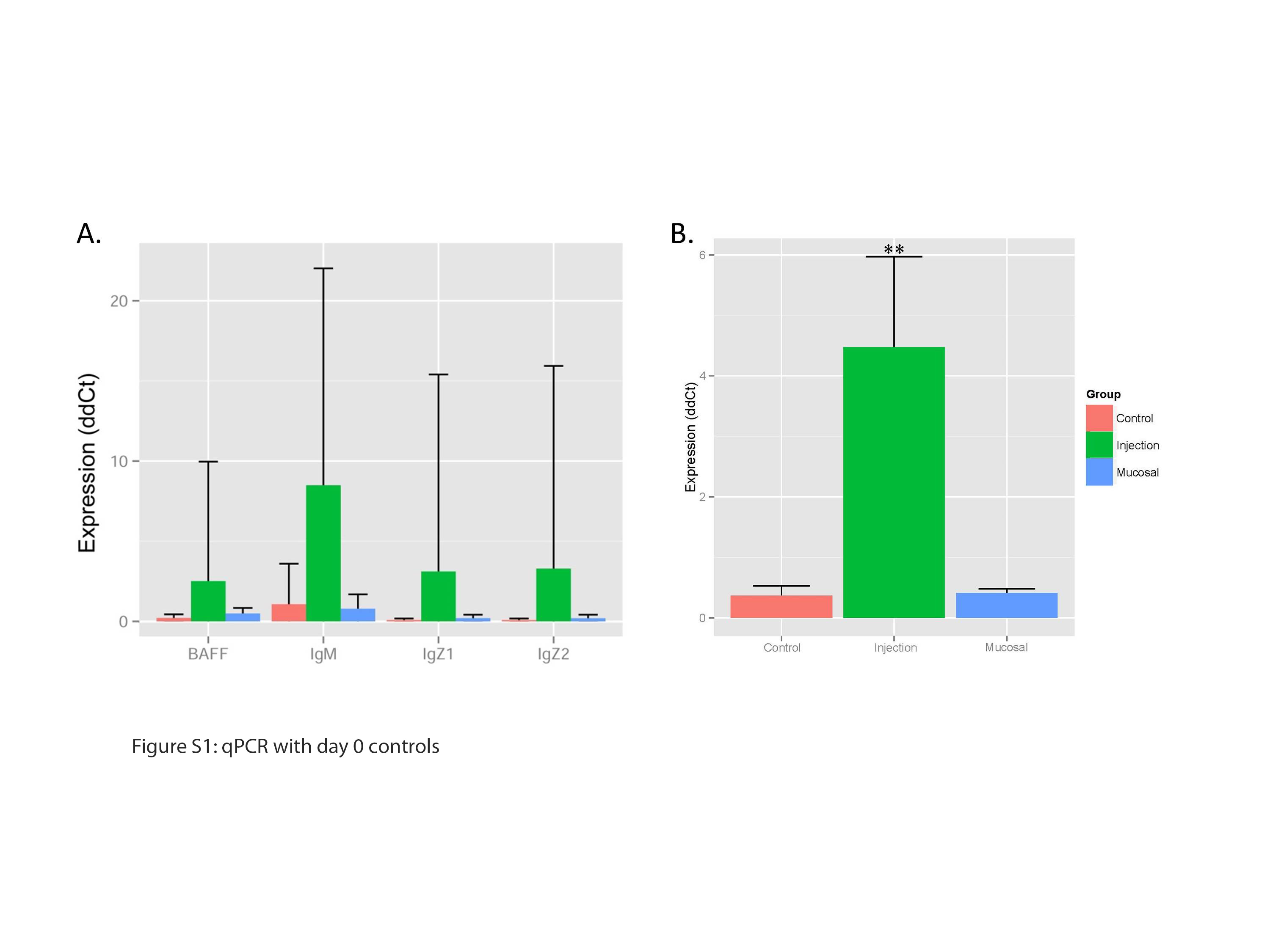

Supplement: Supplementary file 3 [file Image_1.JPEG]
